# Hashimoto's thyroiditis in childhood: presentation modes and evolution over time

**DOI:** 10.1186/1824-7288-39-8

**Published:** 2013-01-30

**Authors:** Filippo De Luca, Simona Santucci, Domenico Corica, Elda Pitrolo, Marika Romeo, Tommaso Aversa

**Affiliations:** 1 Department of Pediatrics, University of Messina, Padiglione NI Policlinico Universitario, Via Consolare Valeria, 98125, Messina, Italy

**Keywords:** Autoimmune thyroid disease, Hashitoxicosis, Subclinical hypothyroidism, Thyroid autoantibodies

## Abstract

Aim of this survey is to report the most recent views about Hashimoto’s thyroiditis (HT) natural history according to the different presentations. In children presenting with either euthyroidism or subclinical hypothyroidism HT spontaneous course is frequently characterized by a trend towards deterioration of thyroid function, whereas in those presenting with overt hyperthyroidism a definitive resolution of hyperthyroid phase is to be expected. Another possible even though unusual outcome of HT is the conversion to Graves’ disease.

## Introduction

Autoimmune thyroid disease (AITD) manifests itself in various clinical forms such as classical Hashimoto' s thyroiditis (HT) and Graves' disease (GD). Although GD and HT have different phenotypes and the mechanisms leading to their dichotomy are unknown, they are generally believed to share a number of common etiological factors. In fact, there have been reports on monozygotic twins in whom one twin had GD and the other one had HT [1-3]. Moreover, both conditions may aggregate in the same families [4] or may even coexist in the same thyroid gland [5], and some individuals may progress from one form to the other. It is more frequent that GD may spontaneously culminate in hypothyroidism due to HT [6], while the development of GD from HT has only occasionally been reported until now [7-9].

HT is the most common form of thyroiditis in childhood [10] and the most frequent cause of pediatric thyroid disease in iodine-replete areas of the world.

Nevertheless, in spite of this high frequency, there are still several concerns and controversies concerning the spontaneous evolution of this condition, at least in childhood.

Aim of the present review is to report the most recent views about HT natural history according to the different presentations.

### HT presentation

At the time of diagnosis, children and adolescent with HT may be asymptomatic, and the main reasons for referral are goiter, hypothyroid symptoms, and findings which occur while working on unrelated problems or for high-risk groups [11]. Thyroid function at presentation may significantly vary in the different pediatric reports [12-16], ranging from euthyroidism to overt hypothyroidism or, occasionally, hyperthyroidism [12]. Further complaints of thyroid function reported in children and adolescent at HT presentation include either subclinical hypothyroidism [13-15], or more rarely, subclinical hyperthyroidism [16]. In a very recent study, we respectively evaluated clinical and laboratory characteristics at HT diagnosis in 608 children and adolescent from three pediatric endocrinology centers in Northern and Southern Italy. The aims of our investigation were to assess the frequency of thyroid function patterns at HT diagnosis and to analyze the factors that may affect the status of thyroid at time of diagnosis [17]. Our test results at presentation showed euthyroidism in 52.1% of patients, overt or subclinical hypothyroidism in 41.4%, and overt or subclinical hyperthyroidism in 6.5%. The mean age of patients with thyroid dysfunctions was significantly lower than that found in euthyroid children. Other variables related to thyroid function patterns were prepubertal status, association with either Down or Turner syndromes, which correlated with increased risk of thyroid dysfunctions, and association with other autoimmune disease, which correlated with decreased risk of thyroid dysfunctions [17]. On overall, thyroid function patterns at HT presentation seem to be mainly conditioned by children age, with an increased risk of severe gland dysfunctions in the cases with early HT presentation [17]. Other factors that may also be involved are the association with either chromosomopathies or other autoimmune disease [17] and environmental factors [18].

The transient hyperthyroid phase of HT is known as hashitoxicosis (Htx), and is believed to result from unregulated release of stored thyroid hormones during inflammatory-mediated destruction of the thyroid gland [19]. Htx has been reported as the second commonest cause of thyrotoxicosis in childhood, after GD [20]. Presenting signs and symptoms of Htx can be very similar to those generally observed in GD, as previously reported in a retrospective study on clinical presentation of Htx in children [21]. Therefore, differential diagnosis of Htx from GD can be particularly challenging when the diagnosis is only based on clinical and biochemical features [22].

### HT evolution over time

According to a very recent prospective study aiming to investigate long-term outcome of HT in the children presenting with overt hyperthyroidism, a definitive resolution of hyperthyroidism is generally observed on average eight months after Htx diagnosis, even though there is a wide variability between subjects [23]. According to that report, management of children with Htx may require a prolonged clinical and biochemical follow-up, but pharmacological treatment is only required in selected cases and non-pharmacological therapies are never needed [23]. Hyperthyroid phase in children with Htx is always followed by definitive resolution, with no relapses and persistent and euthyroidism or hypothyroidism [23].

In children presenting with biochemical and/or clinical euthyroidism, natural history of HT seems to be characterized by a trend towards progressively deteriorating thyroid function in around 50% of cases, whereas at 5 years of follow-up the remaining 50% of cases were reported to remain or become euthyroid [24]. The presence of goiter and elevated thyroglobulin autoantibodies (Abs) at presentation, together with progressive increase in both thyroid peroxydase Abs and TSH may be considered as predictive factors for the future development of hypothyroidism [24].

A similar trend towards a spontaneous deterioration of thyroid function over time has been recently reported in a series of children with HT initially presenting with SH, even though the process is very slow and not predictable in the single case [25]. Therefore, although surveillance is mandatory, it may take a very long time to see whether treatment with L-T_4_ should be implemented or not [25]. The presence of additional risk factors such as celiac disease or elevated TSH and thyroid peroxidase Abs at presentation seems to significantly increase the risk of developing overt hypothyroidism after 3 years in the SH children with HT. Thus, at presentation an increased TSH can be considered as the best predictor of future hypothyroidism from SH, as also suggested by our group [26] and by others [27]. On overall, on the basis of the most recent literature data we can argue that the risk of developing overt hypothyroidism over time is higher in the SH children with an underlying HT than in those with no underlying thyroid disease [28,29]. This inference is strongly supported by the most recent surveys on SH [30-33].

Finally, another possible even though unusual outcome of HT is the conversion to GD [7,34,35]. In fact, according to a recent retrospective epidemiological study, in at least 3,7% of children and adolescents with GD, the onset of hyperthyroidism may be preceded by an HT diagnosis, with either hypothyroidism or euthyroidism [9]. A mechanism that might be hypothesized to account for the change from HT to GD is the alteration in the biological activity of TSH receptor Abs from predominantly thyroid-blocking antibodies during the hypothyroid phase to thyroid-stimulating antibodies when GD manifests itself [8]. However, blocking Abs [8] as a cause of HT are very rare and, therefore, this remains a controversial point, with no good evidence that the change from one disorder to the other really reflects changes in the biological activity of TSH receptor Abs. To sum up, these studies altogether confirm the existence of a possible continuum between HT and GD within the broad spectrum of AITD [7,9,34,35].

## Conclusions

a) In childhood euthyroidism is the most frequent thyroid pattern at HT presentation, followed by either overt or subclinical hypothyroidism and by either overt or subclinical hyperthyroidism; b) presenting thyroid function patterns are mainly conditioned by patients’ age; c) a trend towards progressively deteriorating thyroid function is frequently observed both in the initially euthyroid children and in those presenting with SH; d) the risk of developing overt hypothyroidism over time is higher in the SH children with an underlying HT than in those with no underlying thyroid disease.

## Abbreviations

AITD: Autoimmune thyroid disease; HT: Hashimoto' s thyroiditis; GD: Graves’ disease; Htx: Hashitoxicosis; Abs: Autoantibodies; SH: Subclinical hypothyroidism.

## Competing interests

The authors declare that they have no competing interests.
